# The asymmetry of neural symptoms in Wilson's disease patients detecting by diffusion tensor imaging, resting‐state functional MRI, and susceptibility‐weighted imaging

**DOI:** 10.1002/brb3.930

**Published:** 2018-04-14

**Authors:** Xiang‐xue Zhou, Xun‐hua Li, Ding‐bang Chen, Chao Wu, Li Feng, Jian‐ping Chu, Zhi‐yun Yang, Xin‐bei Li, Haolin Qin, Gui‐dian Li, Hai‐wei Huang, Ying‐ying Liang, Xiu‐ling Liang

**Affiliations:** ^1^ Department of Neurology The East Area of the First Affiliated Hospital Sun Yat‐Sen University Guangzhou China; ^2^ Department of Neurology The First Affiliated Hospital Sun Yat‐Sen University Guangzhou China; ^3^ Department of Radiology The First Affiliated Hospital Sun Yat‐Sen University Guangzhou China; ^4^ Department of Radiology The East Area of the First Affiliated Hospital Sun Yat‐Sen University Guangzhou China

**Keywords:** asymmetry, diffusion tensor imaging, resting‐state functional MRI, susceptibility‐weighted imaging, Wilson's disease

## Abstract

**Objective:**

To investigate the cause of the motor asymmetry in Wilson's disease (WD) patients using functional MRI.

**Methods:**

Fifty patients with WD and 20 age‐matched healthy controls were enrolled. Neurological symptoms were scored using the modified Young Scale. All study subjects underwent diffusion tensor imaging (DTI), susceptibility‐weighted imaging (SWI), and resting‐state functional MRI (rs‐fMRI) of the brain. Six regions of interest (ROI) were chosen. Fiber volumes between ROIs on DTI, corrected phase (CP) values on SWI, amplitude of low‐frequency fluctuation (ALFF), and regional homogeneity (REHO) values on rs‐fMRI were determined. Asymmetry index (right or left value/left or right value) was evaluated.

**Results:**

Asymmetry of rigidity, tremor, choreic movement, and gait abnormality (asymmetry index = 1.33, 1.39, 1.36, 1.40), fiber tracts between the GP and substantia nigra (SN), GP and PU, SN and thalamus (TH), SN and cerebellum, head of the caudate nucleus (CA) and SN, PU and CA, CA and TH, TH and cerebellum (asymmetry index = 1.233, 1.260, 1.269, 1.437, 1.503, 1.138, 1.145, 1.279), CP values in the TH, SN (asymmetry index = 1.327, 1.166), ALFF values, and REHO values of the TH (asymmetry index = 1.192, 1.233) were found. Positive correlation between asymmetry index of rigidity and fiber volumes between the GP and SN, SN and TH (*r* = .221, .133, *p* = .043, .036), and tremor and fiber volumes between the CA and TH (*r* = .045, *p* = .040) was found.

**Conclusions:**

The neurological symptoms of patients with WD were asymmetry. The asymmetry of fiber projections may be the main cause of motor asymmetry in patients with WD.

## 1 INTRODUCTION

Wilson's disease (WD) is an autosomal recessive inherited disorder of copper metabolism, characterized by excessive copper deposition, especially in the liver and brain. Patients with WD have manifold clinical manifestations, MRI changes, and varied responses to treatment (Favrole, Chabriat, Guichard, & Woimant, 2006). The clinical motor symptoms of WD often are asymmetric. Motor asymmetry is seen as having different right or left motor symptoms. There is asymmetry in performance of gait, tremor, and rigidity in patients with WD (Valtteri, 2016). Both the manifestation and extent of motor symptoms on the two sides of patients with WD are quite different. Evaluating the motor asymmetry is an important part of the characterizing neural symptoms of patients with WD. This asymmetry in WD, however, is still part of a mystery. Both the characteristic and mechanism of motor asymmetry are not clear. The asymmetry of neural symptoms may reflect asymmetry of damage to the brain, which may be important feature of pathology process of WD. What we were interested was as follows: (i) the characteristic of asymmetry in neural symptoms of patients with WD and (ii) the cause of the asymmetry. We hypothesized that asymmetry in performance in patients with WD may be a manifestation of asymmetrical metal deposition and damage to both subcortical nuclei and fiber projections between nuclei. To test this, we studied the relationship of asymmetry of neurological symptoms and metal deposition and damage to both subcortical nuclei and fiber projections.

It is hard to find asymmetry in the brain of patients with WD on the conventional MRI. Susceptibility‐weighted imaging (SWI) is superior in its ability to demonstrate metals especially iron and copper than conventional MR imaging (Maija, Hanna, Seppo, Irina, & Prasun, 2013). Corrected phase (CP) values can be used to assess brain metal concentration. Diffusion tensor imaging (DTI) is a method that has been used in recent years to detect changes in white matter integrity (Le Bihan, 2003; Menke et al., 2009; Zhang et al., 2011). DTI metrics include fractional anisotropy (FA), axial diffusivity (λ1), and radial diffusivity (λ2, λ3)^.^(Metwalli et al., 2010) FA and λ values are thought to reflect the condition of axons and myelin (Maija et al., 2013). DTI fiber tracking (FT) can show individual fiber tracts of the brain (Coenen, Mädler, Schiffbauer, Urbach, & Allert, 2011; Mark et al., 2011). DTI can be used to trace subcortical gray matter connections. Resting‐state functional MRI (rs‐fMRI) is a method that has been used to detect functional activity and functional conjunction of the nuclei in the brain.

In this series, we tried to study the characteristic and mechanism of motor asymmetry in patients with WD. We estimated brain metal deposition using SWI to evaluate fiber tracts’ abnormalities using DTI, to evaluate functional activity of the subcortical nuclei using rs‐fMRI, and to investigate the correlations between motor asymmetry and asymmetry of imaging metrics in patients with WD.

## 2 MATERIALS AND METHODS

### 2.1 Patients

The study enrolled 50 patients with WD (28 males and 22 females, mean age 21 ± 4 years) evaluated at the First Affiliated Hospital, Sun Yat‐Sen University, between July 2007 and January 2017. All patients were categorized as cerebral‐type WD, presenting mainly with psychiatric and/or neurological symptoms. The diagnosis of each patient was based on a combination of clinical symptoms and laboratory tests. Clinical indications included decreased serum ceruloplasmin concentration (<0.1 g/L), low serum copper concentration (<0.5 mg/L), elevated 24‐hr urinary copper excretion (>100 μg), and the presence of Kayser–Fleischer (K–F) rings on slit‐lamp examination. Some patients had genetic confirmation (39 patients). As a control group, 20 age‐ and sex‐matched healthy individuals were recruited from the hospital. Informed consent was obtained from all participants prior to enrollment.

The severity of neurological symptoms was assessed using the modified Young Scale (Xiang‐xue, Xun‐hua, & Hai‐wei, 2011). This scale consists of items evaluating dysarthria, dysphagia, rigidity, ataxia, tremor, choreic movement, gait abnormality, and psychogenia. It assesses different aspects of neurological dysfunction due to WD in 16 items: dysarthria (Items 1–2), dysphagia (Items 3–4), rigidity (Items 5–6), ataxia (Items 7–8), tremor (Item 9–10), choreic movement (Item 11–12), gait abnormality (Item 13–14), and psychogenia (Item 15–16). Each item is scored on an ascending 5‐point scale (0–4).

Neurological asymmetry was evaluated as differences in the modified Young Scale scores between the two sides (asymmetry index of neurological symptoms = the modified Young Scale right or left score/left or right score).

### 2.2 Diffusion tensor imaging protocol

All participants underwent DTI tests of the brain. MRI data were obtained using a 1.5 T scanner (Achieva Nova Dual; Philips Healthcare, Best, the Netherlands), equipped with eight‐channel, phase‐array head coils. Conventional MRI sequences included spin‐echo (SE) T1‐weighted images [488 ms repetition time (TR), 15 ms echo time (TE)] obtained in the axial and sagittal planes, with an acquisition time of 2 min, matrix of 256 × 256, and a 250 mm field of view (FOV). Axial T2‐weighted images were also acquired (3,600 ms TR, 100 ms TE). The section thickness was 5 mm. DTI was performed using SE echo‐planar imaging (EPI) (9,500 ms TR, 70 ms TE, 256 × 256 matrix size, 250 mm FOV, 2 mm section thickness with no gaps). Images were obtained with 32‐direction diffusion encoding (b = 1,000 s/mm^2^ for each direction) and with no diffusion encoding (b = 0 s/mm^2^) and were corrected for head and cardiac motion artifacts.

Diffusion tensor imaging data were transferred to a workstation (Philips extended MR workspace 2.6.3.2, Philips Medical Systems) and processed using DTI fiber tracking software for DTI quantitative analysis. ROIs were drawn according to anatomical structures, including the GP, CA, PU, TH, SN, and cerebellum. FA, λ1, λ2, and λ3 values of all ROIs were measured. Fiber tracts were reconstructed using the fiber assignment continuous tracking (FACT) method. Volumes of fiber tracts were counted. Two neuroradiologists separately and independently drew all ROIs on tensor color maps and measured FA and DTI fiber counts. To reduce random variability in the measurements, each value was an average of three different measurements. The reported values are the mean of the two observers.

Asymmetry index of FA = right or left FA values/left or right FA values. Asymmetry index of λ = right or left λ values/left or right λ values. Asymmetry index of fiber volumes = right or left fiber volumes/left or right fiber volumes.

### 2.3 Susceptibility‐weighted imaging protocol

Susceptibility‐weighted imaging images were obtained parallel to the anteroposterior commissural line using a high‐resolution, gradient–echo sequence with following parameters: TR/TE, 60/40 ms; flip angle, 18°; slices, 48; field of view, 230/184 mm; and matrix, 240 × 240. The values of CP of ROIs were checked. Asymmetry index of CP = right or left CP values/left or right CP values.

### 2.4 rs‐fMRI protocol

All study subjects underwent rs‐fMRI of the brain using a 3.0 T scanner (Philips Achieva Nova Dual Plus). The values of amplitude of low‐frequency fluctuation (ALFF) and regional homogeneity (REHO) in ROIs were determined. Asymmetry index of ALFF = right or left ALFF values/left or right ALFF values. Asymmetry index of REHO = right or left REHO values/left or right REHO values.

### 2.5 Statistical analysis

Results were presented as mean ± *SEM*. Statistical analyses were performed using SPSS 13.0. (SPSS Inc., Chicago, IL, USA). Parameters of two sides of brains were compared using independent‐sample *t* tests. Pearson's correlation analysis and regression analysis were used to assess the correlations of MRI parameters with symptom scores. A *p* value < .05 was considered statistically significant.

## 3 RESULTS

### 3.1 Demographic and clinical characteristics of each group

There was no significant difference in gender distribution and age between the patients with WD and healthy volunteers.

### 3.2 Asymmetry of neural symptoms of WD patients

Mean asymmetry index of rigidity, ataxia, tremor, choreic movement, and gait abnormality was 1.33, 1.13, 1.39, 1.36, and 1.40 for patients with WD, respectively.

### 3.3 Asymmetry of MRI parameters of WD patients

Some MRI parameters were different in right and left side in healthy controls. But the difference was not statistical (Table 1).

**Table 1:** MR metrics in patients with WD and the healthy controls

	SN		TH		CA		GP		PU		Cerebellum	
	Left	Right	Left	Right	Left	Right	Left	Right	Left	Right	Left	Right
FA												
WD	0.47 ± 0.20^a^	0.42 ± 0.15^a^	0.37 ± 0.15	0.34 ± 0.12^a^	0.24 ± 0.11	0.28 ± 0.08	0.36 ± 0.14^a^	0.37 ± 0.11^a^	0.26 ± 0.15	0.26 ± 0.13	0.35 ± 0.13^b^	0.30 ± 0.12
Controls	0.63 ± 0.09	0.60 ± 0.09	0.42 ± 0.10	0.42 ± 0.07	0.27 ± 0.10	0.28 ± 0.08	0.50 ± 0.04	0.55 ± 0.03	0.33 ± 0.06	0.35 ± 0.03	0.27 ± 0.13	0.26 ± 0.13
λ1												
WD	1.04 ± 0.34	1.01 ± 0.17	0.91 ± 0.25^b^	1.00 ± 0.33	1.08 ± 0.18	0.99 ± 0.17	0.82 ± 0.21^a^^,^ b	1.17 ± 0.36^a^	0.81 ± 0.51^a^^,^ b	0.95 ± 0.47^a^	0.79 ± 0.30^b^	0.81 ± 0.26
Controls	1.23 ± 0.13	1.17 ± 0.08	1.02 ± 0.11	0.92 ± 0.14	1.05 ± 0.07	1.10 ± 0.15	1.18 ± 0.26	1.40 ± 0.09	1.47 ± 0.07	1.38 ± 0.02	0.87 ± 0.13	0.94 ± 0.13
λ2												
WD	0.78 ± 0.22	0.73 ± 0.22	0.73 ± 0.14	0.78 ± 0.19	0.82 ± 0.24	0.84 ± 0.17	0.82 ± 0.16^a^^,^ b	0.98 ± 0.34^a^	1.14 ± 0.44^a^	1.04 ± 0.2^a^	0.62 ± 0.23	0.71 ± 0.27
Controls	0.77 ± 0.14	0.74 ± 0.08	0.74 ± 0.09	0.76 ± 0.13	0.76 ± 0.21	0.88 ± 0.14	0.64 ± 0.12	0.74 ± 0.05	0.72 ± 0.05	0.81 ± 0.05	0.54 ± 0.13	0.65 ± 0.13
λ3												
WD	0.51 ± 0.23	0.46 ± 0.22	0.48 ± 0.19	0.53 ± 0.16	0.64 ± 0.17	0.63 ± 0.15	0.56 ± 0.18^b^	0.65 ± 0.26	0.87 ± 0.35^a^	0.84 ± 0.22^a^	0.46 ± 0.25	0.54 ± 0.26
Controls	0.41 ± 0.07	0.46 ± 0.09	0.40 ± 0.11	0.41 ± 0.09	0.65 ± 0.13	0.54 ± 0.13	0.56 ± 0.10	0.57 ± 0.05	0.50 ± 0.07	0.49 ± 0.04	0.48 ± 0.13	0.57 ± 0.13
CP												
WD	1,817 ± 47^a^^,^ b	1,837 ± 52^a^	2,054 ± 21^a^^,^ b	2,075 ± 24^a^	2,024 ± 36^a^	2,013 ± 41^a^	1,996 ± 37	1,989 ± 42	2,013 ± 52^a^	2,009 ± 39^a^	2,093 ± 27^a^	2,080 ± 46^a^
Controls	2,019 ± 77	2,015 ± 63	2,132 ± 32	2,129 ± 41	2,085 ± 45	2,088 ± 62	2,013 ± 69	2,019 ± 52	2,063 ± 23	2,071 ± 51	2,155 ± 19	2,169 ± 21
ALFF												
WD			6.81 ± 3.34^b^	5.51 ± 3.76^a^	5.05 ± 3.67^a^	5.25 ± 3.51^a^	6.70 ± 0.78	6.76 ± 1.08	6.35 ± 0.82^a^	6.55 ± 1.06^a^		
Controls			7.56 ± 0.50	7.85 ± 0.54	8.22 ± 0.62	8.23 ± 0.65	7.01 ± 0.79	6.98 ± 0.77	7.30 ± 0.81	7.40 ± 0.72		
REHO												
WD			6.08 ± 3.39^a^^,^ b	7.27 ± 3.02	6.16 ± 3.34^a^	6.19 ± 3.41	7.41 ± 0.98	7.23 ± 1.90	7.48 ± 1.86	7.68 ± 1.83		
Controls			8.17 ± 0.88	7.87 ± 2.20	8.41 ± 0.73	7.31 ± 2.83	7.77 ± 0.65	7.65 ± 2.17	7.44 ± 2.04	6.72 ± 3.24		

ALFF, amplitude of low‐frequency fluctuation; PU, putamen; GP, globus pallidus; CA, head of the caudate nucleus; TH, thalamus; SN, substantia nigra; REHO, regional homogeneity; WD, Wilson's disease.

Results reported as mean±SEM.

a *p* ≤ .05 compared with healthy controls.

b *p* ≤ .05 compared with the right side.

On comparing the DTI metrics of the two hemispheres in patients with WD, significantly different λ1, λ2, and λ3 values in the GP, λ1 values in PU, and FA values of the cerebellum were observed (Tables 1 and 2).

**Table 2:** Asymmetry index and *p* values of Wilson's disease patients

	FA	λ1	λ2	λ3	CP	ALFF	REHO
PU							
Asymmetry index	1.01 ± 0.13	1.17 ± 0.23	1.11 ± 0.11	1.05 ± 0.22	1.01 ± 0.17	1.03 ± 0.12	1.03 ± 0.15
*p*	.093	.032^a^	.174	.338	.725	.071	.507
GP							
Asymmetry index	1.02 ± 0.34	1.43 ± 0.12	1.20 ± 0.04	1.16 ± 0.22	1.01 ± 0.31	1.00 ± 0.30	1.02 ± 0.22
*p*	.077	.032^a^	.042^a^	.046^a^	.642	.175	.056
CA							
Asymmetry index	1.14 ± 0.22	1.09 ± 0.07	1.02 ± 0.21	1.01 ± 0.07	1.00 ± 0.21	1.04 ± 0.26	1.00 ± 0.11
*p*	.236	.180	.076	.115	.761	.680	.853
TH							
Asymmetry index	1.08 ± 0.14	1.10 ± 0.09	1.07 ± 0.22	1.11 ± 0.15	1.00 ± 0.04	1.24 ± 0.10	1.20 ± 0.29
*p*	.420	.271	.091	.904	.048^a^	.036^a^	.040^a^
SN							
Asymmetry index	1.12 ± 0.33	1.03 ± 0.15	1.07 ± 0.39	1.11 ± 0.12	1.00 ± 0.27		
* p*	.339	.609	.073	.178	.044^a^		
Cerebellum							
Asymmetry index	1.17 ± 0.16	1.03 ± 0.17	1.15 ± 0.30	1.17 ± 0.16	1.00 ± 0.13		
* p*	.047^a^	.163	.873	.093	.430		

ALFF, amplitude of low‐frequency fluctuation; PU, putamen; GP, globus pallidus; CA, head of the caudate nucleus; TH, thalamus; SN, substantia nigra; REHO, regional homogeneity.

Results reported as mean ± *SEM*. Asymmetry index: right or left values/left or right values.

a *p *≤* *.05 on comparing the metrics of the two hemisphere using independent‐sample *t* tests.

**Figure 1:**
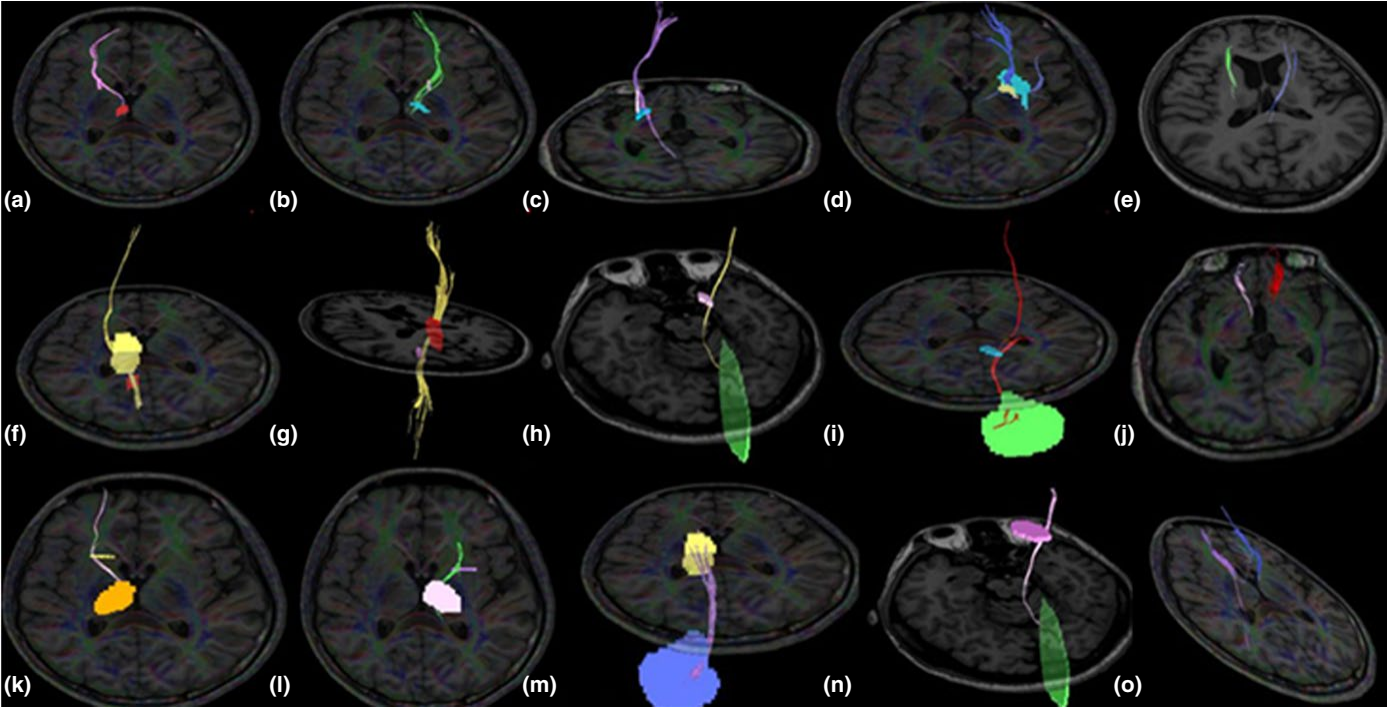
Diffusion tensor imaging results showing the volumes of fiber tracts between ROIs in the brains of patients with WD. The volumes of the left (b) and right (a) fibers between the GP and SN differed (*p* = .043, asymmetry index = 1.233), the volume of fibers between the GP and PU was lower on the right side (c) than on the left side (d) (*p* = .035, asymmetry index = 1.413), the volumes of the left and right fibers between the PU and SN differed (e) (*p* = .044, asymmetry index = 1.260), the volume of fibers between the SN and TH was lower on the right side (f) than on the left side (g) (*p* = .046, asymmetry index = 1.220), the volume of fibers between the SN and cerebellum was higher on the right side (h) than on the left side (i) (*p* = .049, asymmetry index = 1.437), the volumes of the left and right fibers between the CA and SN differed (j) (*p* = .023, asymmetry index = 1.503), the volume of fibers between the CA and TH was higher on the right side (k) than on the left side (l) (*p* = .037, asymmetry index = 1.145), the volume of fibers between the TH and cerebellum was higher on the right side (m) than on the left side (n) (*p* = .041, asymmetry index = 1.279), the volumes of the left and right fibers between the PU and CA differed (O) (*p* = .016, asymmetry index = 1.138). Abbreviations: PU, putamen; ROI, regions of interest; GP, globus pallidus; CA, head of the caudate nucleus; TH, thalamus; SN, substantia nigra; WD, Wilson's disease

In patients with WD, the volumes of fiber tracts between the GP and SN, GP and PU, PU and SN, SN and TH, SN and cerebellum, CA and SN, PU and CA, CA and TH, and TH and cerebellum differed significantly between the two hemispheres (Figure 1).

**Figure 2:**
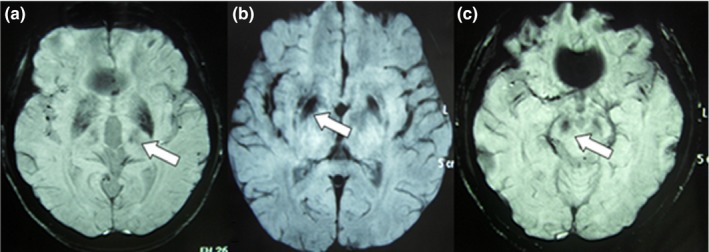
Susceptibility‐weighted imaging results showing low signals of ROIs in the brains of patients with WD. CP values of the TH was lower on the left side than on the right side (a), CP values of the PU were lower on the right side than on the left side (b), CP values of the SN were lower on the right side than on the left side (c). Abbreviations: PU, putamen; TH, thalamus; SN, substantia nigra; ROI, regions of interest; WD, Wilson's disease

In patients with WD, a significant difference in CP values between the two hemispheres was observed in the TH and SN (Tables 1 and 2, Figure 2).

**Figure 3:**
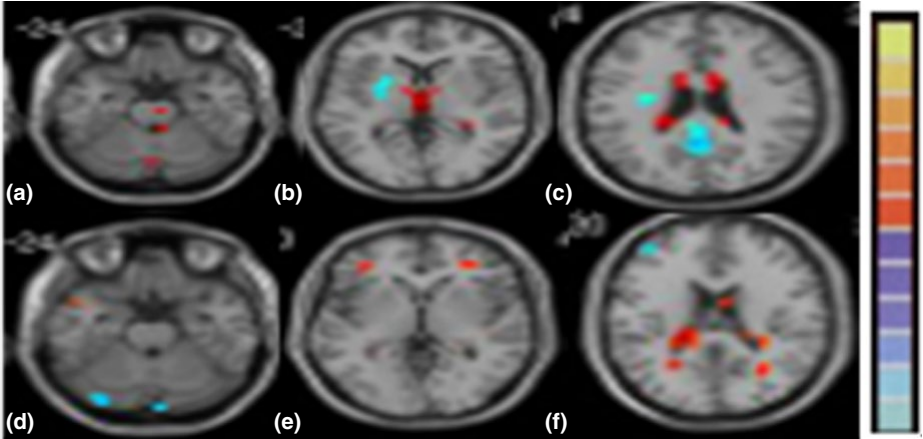
rs‐fMRI results of ROIs in the brains of patients with WD. Blue signals show the lower ALFF values (a–c) or REHO values (d–f), while red signals show higher ALFF values or REHO values compared with normal controls. ALFF values of the TH (b), REHO values of the TH on the two hemisphere differed (e). Abbreviations: TH, thalamus; ALFF, amplitude of low‐frequency fluctuation; REHO, regional homogeneity; ROI, regions of interest; WD, Wilson's disease

In patients with WD, the left and right ALFF values and REHO values of the TH differed significantly (Tables 1 and 2, Figure 3).

### 3.4 Relationship between symptoms asymmetry and MR asymmetry

On analyzing the relationship between symptoms asymmetry and MR asymmetry using regression analysis, the influencing factors of rigidity asymmetry were as follows: asymmetry of λ1 values in the GP, asymmetry of fiber volumes between the GP and SN, SN and TH, and SN and cerebellum. The influencing factors of tremor asymmetry were as follows: asymmetry of fiber volumes between the CA and TH, and TH and cerebellum. The influencing factors of choreic movement asymmetry were as follows: asymmetry of fiber volumes between the CA and TH. The influencing factors of gait abnormality were as follows: asymmetry of fiber volumes between the GP and SN, and SN and TH (Table 3).

**Table 3:** Influencing factors of neurological asymmetry in Wilson's disease patients

	Factors	B	*p*
Rigidity asymmetry index	Asymmetry index of λ1 values in the GP	0.33	.042
	Asymmetry index of fiber volumes between the GP and SN	13.01	.031
	Asymmetry index of fiber volumes between the SN and TH	2.79	.029
	Asymmetry index of fiber volumes between the SN and cerebellum	0.27	.035
Tremor asymmetry index	Asymmetry index of fiber volumes between the CA and TH	8.20	.041
	Asymmetry index of fiber volumes between the TH and cerebellum	4.15	.029
Choreic movement asymmetry index	Asymmetry index of fiber volumes between the CA and TH	19.01	.026
Gait abnormality index	Asymmetry index of fiber volumes between the GP and SN	2.12	.043
	Asymmetry index of fiber volumes between the SN and TH	1.16	.033

Pearson's correlation analysis showed positive correlation between the asymmetry index of rigidity and the asymmetry index of fiber volumes between the GP and SN (*r* = .221, *p* = .043), and asymmetry index of conjunction fiber volumes between the SN and TH (*r* = .133, *p* = .036). Positive correlation was found between the asymmetry index of tremor and the asymmetry index of conjunction fiber volumes between the CA and TH (*r* = .045, *p* = .040).

We found no correlation between symptom asymmetry and asymmetry of CP values, and asymmetry of rs‐fMRI parameters, respectively.

## 4 DISCUSSION

Motor asymmetry is defined as having predominant right or left motor symptoms (Esther, Pablo, & Jesus, 2010). A few investigations described the notion of asymmetric motor performance in WD (Esther et al., 2010). We also found the asymmetry of neurological symptoms in patients with WD. However, the characteristic and mechanism of motor asymmetry of patients with WD are still unclear. In this series, we tried to estimate the motor asymmetry of patients with WD and investigate the cause of motor asymmetry using MR methods.

Motor asymmetry is common in neurodegenerative diseases. Parkinson's disease (PD) typically manifests with asymmetric motor symptom onset (Huang et al., 2007). In our series, the asymmetry of gait, rigidity, tremor, and choreic movement was common in patients with WD. The motor asymmetry may be useful in the clinical diagnosis of WD. And it is important to evaluating the motor asymmetry when estimating the neural symptoms of patients with WD in clinical works.

The mechanism of asymmetry is still unclear. PD asymmetry may be related to the asymmetric loss of dopaminergic neurons in the substantia nigra (Esther et al., 2010). But the contralateral dopaminergic loss that mirrors lateralized motor symptoms may be insufficient (Valtteri, 2016). There is recent work to suggest that a proportion of PD patients do not present their primary symptoms on the side of the body contralateral to the predominant dopamine deficit (Valtteri, 2016). Some investigations found that asymmetry of movements may originate from higher levels of the central nervous system (Johnsen, Mogensen, Sunde, & Østergaard, 2009). WD is an autosomal recessive defect of cellular copper export. Neurological symptoms of WD is secondary to damage to networks of the extracorticospinal tract, which includes both nuclei and fiber projections (Jadav et al., 2013; King, Walshe, Kendall, Halligan, & Hall‐Craggs, 1996; Prashanth et al., 2010; Xiang‐xue et al., 2016a). The motor asymmetry in WD may be related to asymmetry of copper deposition and damage to extracorticospinal tract. To test this hypothesis, we tried to analyze the correlation between asymmetry of motor performance and metal deposition, and brain damage in patients with WD. In this series, we used functional imaging to evaluate metal deposition and damage to the brain. We found that MRI parameters were not statistically different in right and left side in healthy controls. The asymmetry of MRI parameters found in patients with WD was caused by damage of the disease.

Diffusion tensor imaging, a method of visualizing white matter fibers in vivo, has applications in neurodegeneration (Konishi et al., 2005). It is thought that the value of FA could reflect the integrity of myelin and neuraxons, while axial (λ1) and radial (λ2 and λ3) diffusivities may be markers of axonal damage and demyelination, respectively (Metwalli et al., 2010). Moreover, loops of the extracorticospinal tract can be reconstructed by FACT. In this study, we found asymmetry of FA and λ values in the subcortical nuclei, indicating the asymmetric axonal damage and demyelination in nuclei of patients with WD. Meanwhile, we observed asymmetry of fiber projections between nuclei, indicating that damage to the extracorticospinal network was not symmetrical. We found correlation between rigidity asymmetry and DTI metrics asymmetry in the GP, indicating that the asymmetry of damage to the GP may be correlated with rigidity asymmetry in patients with WD. Otherwise, the asymmetry of neurological symptoms may result from the asymmetry of damage to fiber connections. We observed that the rigidity asymmetry correlated with the asymmetry of fiber projections in SN, tremor asymmetry correlated with the asymmetry of fiber projections in TH, cerebellum, and CA. Compared with the asymmetry of damage to the nuclei, the asymmetry of fiber projections may have more influence on motor asymmetry.

The hypointense signals in SWI can reflect mineral deposition^.^(Jae‐Hyeok et al., 2012). It is possible that the observed abnormalities may reflect both the copper and iron deposition in patients with WD (Hingwala, Kesavadas, Thomas, & Kapilamoorthy, 2013). The negative correlation between CP values and mineral accumulation was confirmed (Zhang et al., 2009). In this study, we found asymmetry of CP values in subcortical nuclei as SN and TH, indicating the mineral deposition was not symmetrical in the brains of patients with WD. In the previous researches, the correlations between CP values and neurological symptoms were found. The correlations between CP values of the SN and UPDRS motor scores indicate that the amount of iron deposition in the SN might reflect the severity of PD (Yong‐Hee et al., 2013). CP values of the GP, PU, and SN have been shown to be associated with the extent of tremor (Maija et al., 2013). The correlation between the extent of abnormal posture and the anterior GP has been found (Maija et al., 2013). In contrast to others, we did not find correlation between the asymmetry of CP values in subcortical nuclei and the motor asymmetry in patients with WD. It may be not the metal deposition, but the asymmetric damage to the brain is involved in neurological symptom asymmetry in patients with WD.

Rs‐fMRI is a method used to detect functional activity of nuclei in the brain. The value of ALFF and REHO could reflect the spontaneous movement and cooperativity of neurons in the brain separately. We found decreased ALFF and REHO values in the subcortical nuclei of patients with WD, indicating the decreased spontaneous movement and the cooperativity of neurons of these nuclei. The asymmetry of ALFF and REHO values in nuclei of patients with WD was found in this study. The functional movements of neurons in the brains of patients with WD were also not symmetrical. In our previous research, the correlation between rs‐fMRI metrics of subcortical nuclei and psychiatric symptoms was found in patients with WD (Xiang‐xue et al., 2016b). However, we did not find correlation between the asymmetry of rs‐fMRI metrics and the motor asymmetry in patients with WD. The asymmetry of functional movements of neurons in the brain may be not the cause of the motor asymmetry in patients with WD.

### 4.1 Limitations

This study had several limitations. First, as ROIs were drawn by radiologists relative to anatomical structures, there may have been random variability in the measurements. To reduce this variability, all measurements were made by two neuroradiologists independently, with the value for each being an average of three different measurements. Furthermore, fiber tracts were reconstructed using the FACT method, which can reconstruct fiber projections between pairs of ROIs, but cannot distinguish between outflow and inflow fiber tracts. This made it difficult to define the network path in the brain using DTI. Third, SWI is not a true quantitative method to measure brain mineral. More researches to evaluate the correlation between pathology of brains and neurological symptoms should be performed in the future.

## 5 CONCLUSION

The neurological symptoms of patients with WD were not symmetrical. Meanwhile, we observed asymmetry of DTI, SWI, and rs‐fMRI metrics in patients with WD, indicating the asymmetry of fiber projections, metal deposition, and functional movements of neurons. Compared with the asymmetry of metal deposition and the functional movements of neurons, the asymmetrical damage to fiber projections may be the main cause of motor asymmetry in patients with WD.

## CONFLICT OF INTEREST

None declared.
